# µ-opioid receptor-mediated downregulation of midline thalamic pathways to basal and central amygdala

**DOI:** 10.1038/s41598-019-54128-8

**Published:** 2019-11-28

**Authors:** L. Goedecke, X. Bengoetxea, P. Blaesse, H.-C. Pape, K. Jüngling

**Affiliations:** Institute of Physiology I, Westfaelische Wilhelms-Universitaet Muenster, Muenster, Germany

**Keywords:** Neural circuits, Neurotransmitters

## Abstract

Brain µ-opioid receptors (MOR) mediate reward and help coping with pain, social rejection, anxiety and depression. The dorsal midline thalamus (dMT) integrates visceral/emotional signals and biases behavior towards aversive or defensive states through projections to the amygdala. While a dense MOR expression in the dMT has been described, the exact cellular and synaptic mechanisms of µ-opioidergic modulation in the dMT-amygdala circuitry remain unresolved. Here, we hypothesized that MORs are important negative modulators of dMT-amygdala excitatory networks. Using retrograde tracers and targeted channelrhodopsin expression in combination with patch-clamp electrophysiology, we found that projections of dMT neurons onto both basal amygdala principal neurons (BA PN) and central amygdala (CeL) neurons are attenuated by stimulation of somatic or synaptic MORs. Importantly, dMT efferents to the amygdala drive feedforward excitation of centromedial amygdala neurons (CeM), which is dampened by MOR activation. This downregulation of excitatory activity in dMT-amygdala networks puts the µ-opioid system in a position to ameliorate aversive or defensive behavioral states associated with stress, withdrawal, physical pain or social rejection.

## Introduction

The brain’s µ-opioid system has long been known for its important analgesic effects and its role in addiction and reward^1^. It is further central to regulating appetite, reproduction, social rejection, mood and anxiety^2,3^. Generally, µ-opioids are described to relieve physical as well as social pain and to alleviate aversive states as they have antidepressant-like and anxiolytic-like effects^1–4^. A candidate region for mediating some of the µ-opioidergic effects is the dorsal midline thalamus (dMT) with its exceptionally high µ-opioid receptor (MOR) density^5–7^. The dMT is composed of the paraventricular (PVT) and paratenial (PT) nucleus of the thalamus^8,9^ and receives the vast majority of inputs from brainstem, hypothalamus and prefrontal cortex^8,10^. Efferent projections of the dMT innervate the Nucleus accumbens (NAc), bed nucleus of the stria terminalis (BNST) and central amygdala (CeA), with weaker projections to prefrontal cortical regions and the basal amygdala (BA)^9,11,12^. The dMT is considered an important switch between input and output signals, integrating signals of positive and negative valence related to visceral or emotional states and influencing key brain regions involved in behavioral regulation. It is central to mediating arousal, wakefulness, and reward as well as aversive states associated with stress, anxiety, drug withdrawal, social rejection and pain^4,9,11–16^. Approach-avoidance behavior is thought to be mediated by a dMT-NAc pathway, while dMT-amygdala circuits have been related to aversive or defensive behavioral states^8^. The amygdala has long been implicated in conditioned behavior, especially fear, and projections of BA principal neurons (BA PN) to the centromedial amygdala (CeM) signal negative emotional valence^17^. Centromedial amygdala neurons mediate freezing, a stereotypical defensive behavioral response in mice^18^. A hypothetical dMT-BA-CeM excitatory circuit, however, has not been placed in a specific behavioral context. More particularly, the exact cellular and synaptic mechanisms of µ-opioidergic modulation in this dMT-amygdala circuit remain unresolved. At the cellular level, ligand-binding to MORs activates G-protein-coupled inwardly rectifying potassium channels (GIRKs) leading to membrane hyperpolarization^19,20^. It has been proven that neurons in the dMT are inhibited by MOR signaling via this mechanism^21^. In addition, MORs mediate the inhibition of N-, P/Q- and R-type voltage-activated calcium channels^20^, whereby µ-opioids may regulate synaptic transmitter release. Here, using a retrograde tracer in the amygdala or targeted expression of channelrhodopsin in the dMT in combination with patch-clamp electrophysiology, we provide a detailed characterization of the µ-opioidergic neuromodulatory impact on a specific dMT-amygdala circuitry. The results reveal that MOR stimulation in the dMT inhibits both BA and CeL projecting dMT neurons somatically. At the synaptic level, excitatory transmission of dMT neurons onto BA neurons is attenuated. As BA PNs provide considerable excitatory input to the centromedial amygdala (CeM), we investigated whether the dMT drives feedforward excitation of centromedial amygdala (CeM) neurons and found it to be downregulated by MOR activation. Interestingly, dMT input to the CeL was sparse as compared to BA and µ-opioidergic inhibition of synaptic transmission was significantly weaker as compared to dMT-BA synapses.

## Results

### MOR stimulation inhibits both BA and CeL projecting dMT neurons

**Figure 1:**
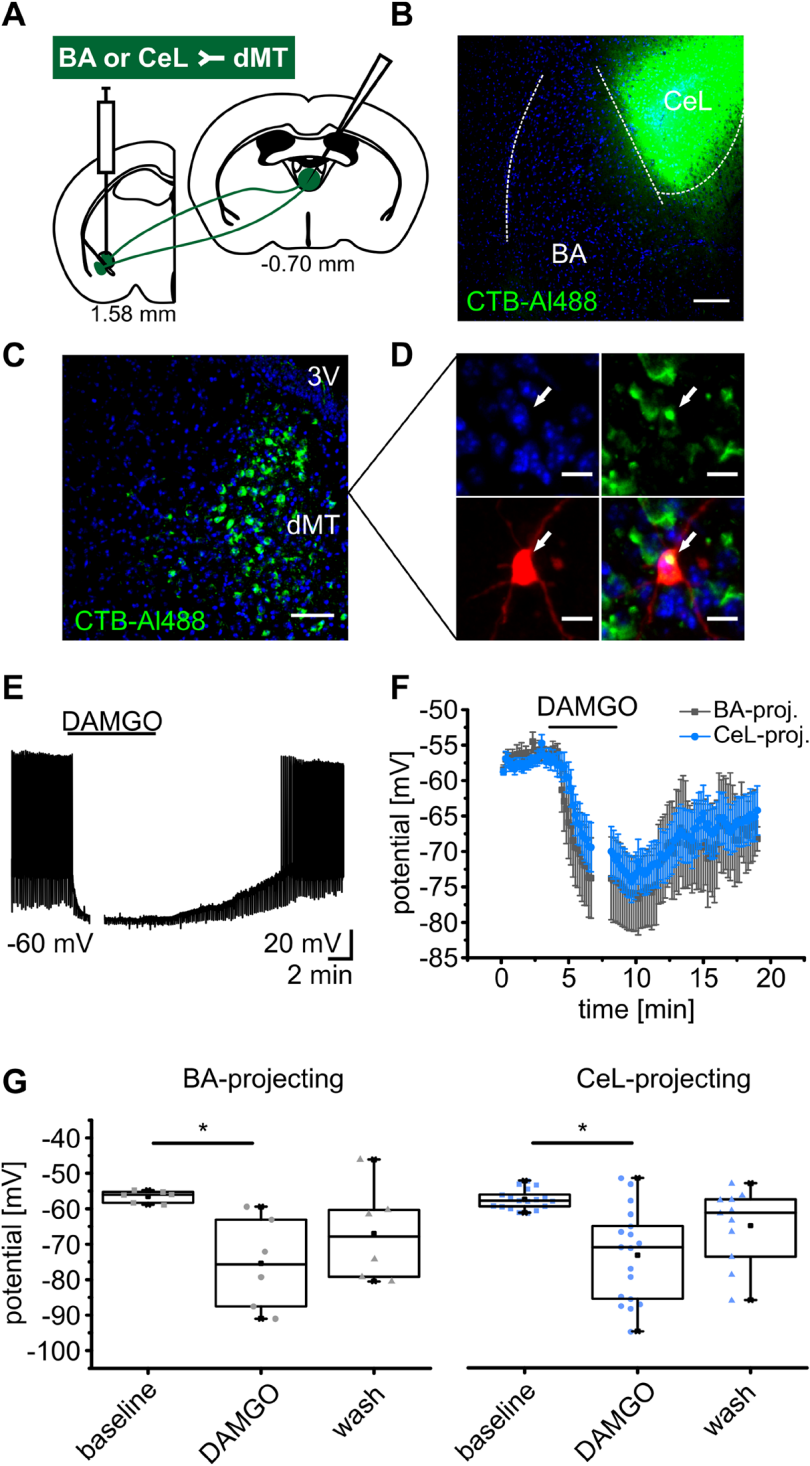
MOR stimulation inhibits both BA- and CeL-projecting dMT neurons. (**A**) Delivery of the retrograde tracer CTB-Al488 either into CeL or BA. Targeting of retrogradely labeled neurons in the dMT for whole-cell patch-clamp recordings. (**B**) Example of a CTB-Al488 injection into the CeL. Scale bar represents 100 µm. (**C**) Retrogradely labeled somata in the dMT. Scale bar represents 50 µm. (**D**) *Post-hoc* identification of a retrogradely CTB-Al488 (green) labeled dMT neuron filled with neurobiotin (red) during the recording indicated by a white arrow. Cell nuclei are labeled with DAPI (blue) and all channels are merged in the overlay. Scale bar represents 10 µm. (**E**) Representative trace of a CeL-projecting dMT neuron recorded in current-clamp mode showing a significant hyperpolarization in response to DAMGO. Period of substance application is indicated by the horizontal line and leads to interruption of spiking. The gap in the trace indicates the period during maximal effect when the cells were manually clamped back to approximately −60 mV in order to determine the apparent input resistance excluding contributions of voltage-dependent conductances. (**F**) Time course of the mean membrane potential in response to DAMGO, averaged from recordings in CeL- (blue) and BA-projecting (grey) dMT neurons. (**G**) Quantification of the membrane potential of CeL- (n = 18 cells/6 mice) and BA-projecting (n = 6 cells/4 mice) dMT neurons, before (baseline), during maximal DAMGO effect and following wash.

There is evidence for strong MOR expression in the dMT (Supplementary Fig. S1A)^5–7^ and dMT neurons projecting to BA and CeL seem to constitute non-overlapping populations^22^. In a first line of experiments we thus sought to identify differences in somatic responses to MOR stimulation in the populations of dMT neurons with projections to either BA or CeL. In order to identify BA and CeL projecting dMT neurons, a cholera toxin subunit B Alexa Fluor 488 conjugate (CTB-Al488) was stereotaxically injected bilaterally into either CeL or BA and retrogradely labeled neurons were identified in the dMT (Fig. 1A–D). These neurons were targeted for whole-cell patch-clamp recordings and the MOR agonist DAMGO was bath applied for 5 min during the recording. MOR activation by 250 nM DAMGO transiently hyperpolarized CeL projecting dMT neurons recorded in current-clamp mode (Fig. 1E,F). The mean membrane potential was at −57.5 ± 1 mV during baseline, −73.2 ± 3 mV in the presence of DAMGO, and −65 ± 3 mV after wash out (one way ANOVA: F_(2,44)_ = 12.2, p = 6.3E-5; *post hoc* test: baseline vs DAMGO, p = 3.7E-5) (Fig. 1G). Similarly, BA projecting dMT neurons were hyperpolarized from −56.5 ± 1 mV to −75.4 ± 5 mV by DAMGO application. After wash, the mean membrane potential returned to a more depolarized potential of −67.0 ± 5 mV (one way ANOVA: F_(2,15)_ = 4.6, p = 0.027; *post hoc* test: baseline vs DAMGO, p = 0.025) (Fig. 1G). 89% of CeL- and 100% of BA-projecting dMT neurons showed a significant hyperpolarization in response to DAMGO. Furthermore, the apparent input resistance of CeL- and BA-projecting dMT neurons was reduced by DAMGO application. To characterize the influence of DAMGO on the input resistance, dMT neurons were manually clamped back to −60 mV while the DAMGO effect on the membrane potential was maximal. This was done in order to isolate direct DAMGO-induced effects and to exclude voltage-dependent currents contributing to changes in input resistance. Since the effect size in CeL-projecting dMT neurons was not significantly different from the effect size of DAMGO in BA-projecting dMT neurons, the data of the two groups were pooled. The mean input resistance of CeL/BA-projecting dMT neurons amounted to 639 ± 19 MΩ under baseline, significantly decreased to 502 ± 36 MΩ in presence of DAMGO and returned to 567 ± 52 MΩ after wash (one way ANOVA: F_(2,54)_ = 4.778, p = 0.012; *post hoc* test: baseline vs DAMGO, p = 9.53E-3) (Supplementary Fig. S1B). Together, the activation of MORs leads to a prominent hyperpolarization and thus, inhibition of both CeL- and BA-projecting neurons in the dMT and this effect was accompanied by a reduction in membrane input resistance. Since synaptic blockers did not abolish these effects, they are likely to be mediated by somatic MORs which corresponds well with the existing literature^20,21,23^.

### Increased glutamatergic transmission at dMT-BA synapses as compared to dMT-CeL synapses

**Figure 2:**
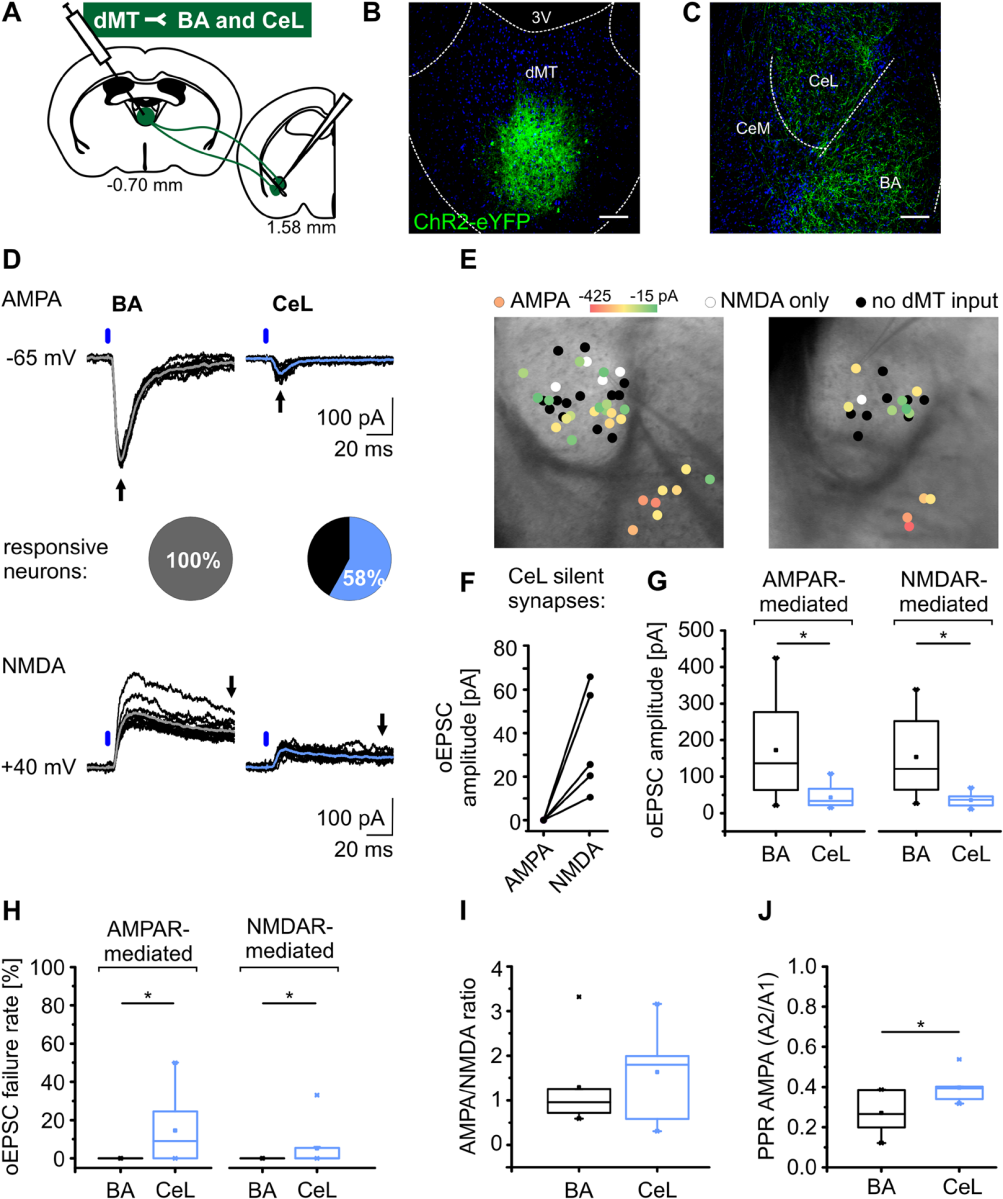
Increased glutamatergic transmission at dMT-BA synapses as compared to dMT-CeL synapses. (**A–C**) dMT neurons were transduced with AAV2-ChR2-eYFP and optogenetic experiments were carried out 5-7 weeks following surgery. dMT projections in the amygdala were light stimulated and patch-clamp recordings were performed on BA PNs and CeL neurons. (**A**) Schematics of the experimental approach, (**B**) histological verification of the dMT injection site, and (**C**) of ChR2-eYFP expressing dMT projections in the amygdala. Scale bars represent 100 µm. (**D**) oEPSCs evoked by light-stimulation of dMT afferents in BA (grey) and CeL (blue) neurons. Original traces (overlay of 10 oEPSCs from a single neuron and the average in grey/blue) of responses evoked at −65 mV exemplify larger oEPSC amplitudes in BA compared to CeL neurons. Responses obtained are considered AMPAR-mediated and the amplitude was determined from peak responses (arrows in upper original trace; overlay and average as above). NMDAR-mediated components were determined from responses evoked at +40 mV 95 ms after stimulus onset (arrows in lower original traces). Blue bars indicate the light stimulus. oEPSCs could be evoked in all recorded BA PNs (n = 12 out of 12 cells/4 mice), but only in 58% of CeL neurons (n = 29 out of 50 cells/5 mice). (**E**) Localization of all individual recorded neurons in the CeL or BA. Color codes for the AMPAR-mediated oEPSC amplitude (from −425 pA in red to −15 pA in green). Neurons without AMPAR- but with NMDAR-mediated input are displayed in white, neurons receiving neither AMPAR-mediated nor NMDAR-mediated input are marked in black. (**F**) Some CeL neurons (n = 5 out of 50 cells/5 mice) show only NMDAR-mediated but no AMPAR-mediated oEPSCs indicating silent synapses. (**G,H**) Quantification of the absolute amplitude (**G**) and failure rate (**H**) of AMPAR- and NMDAR-mediated oEPSCs in CeL (AMPA n = 24 cells/5 mice, NMDA n = 16 cells/3 mice) and BA neurons (AMPA n = 12 cells/4 mice, NMDA n = 10 cells/3 mice). (**I,J**) AMPA/NMDA ratio (**I**) (BA n = 10 cells/3 mice, CeL n = 14 cells/3 mice) and paired-pulse ratio (**J**) (BA n = 6 cells/2 mice, CeL n = 6 cells/3 mice) at dMT-BA and dMT-CeL synapses.

Since MOR-mediated somatic hyperpolarization was indistinguishable in the populations of dMT neurons projecting to either BA or CeL, we went on to investigate these projections at the synaptic level in the target areas. In view of the relative lack of electrophysiological data on dMT-CeL and dMT-BA synapses, we characterized basic properties of dMT-CeL versus dMT-BA excitatory transmission. Mice were stereotaxically injected with AAV2-ChR2-eYFP into the dMT (Fig. 2A–C). Viral injection sites in the dMT were similar for all five mice included in the analysis (Supplementary Fig. S2A,B). Notably, dMT projections were predominantly localized to the anterior part of the BA (aBA) (Supplementary Fig. S2C). Optical stimulation of dMT projections in the amygdala (Fig. 2C, Supplementary Fig. S2B) was set to 1 ms pulse duration and 14 mW intensity, which reliably evoked excitatory postsynaptic currents (oEPSCs) in both BA and CeL neurons recorded at −65 mV in voltage-clamp mode (Fig. 2D). The events can be considered AMPA receptor (AMPAR)-mediated oEPSCs, as current flow through NMDA receptors (NMDAR) at −65 mV is negligible due to its internal block by magnesium. To relieve the magnesium block of NMDARs, the same BA and CeL neurons were then clamped at +40 mV and the amplitude of NMDAR-mediated oEPSCs was analyzed 95 ms after the light stimulus, where the AMPAR component had declined to zero. Further, all recordings were done in the presence of GABA_A/B_ receptor antagonists as well as Tetrodotoxin (TTX) and 4-Aminopyridine (4-AP) to select for glutamatergic, monosynaptic inputs^24^. In order to compare neuronal properties and regional distribution in the amygdala, the localization of all recorded CeL and BA neurons and their dMT synaptic input is summarized in Fig. 2E. While 100% of recorded BA PNs received AMPAR- and/or NMDAR-mediated excitatory input from dMT, it was only 58% of recorded neurons in the CeL (Fig. 2D,E). The quantification revealed that AMPAR-mediated oEPSCs were significantly larger in BA as compared to CeL neurons with a mean amplitude of 172.76 ± 37.44 pA and 44.12 ± 5.48 pA, respectively (unpaired t-test: p = 5.6E-3) (Fig. 2G). Means were calculated from all oEPSC success amplitudes in 10-20 light stimulations, while events smaller than 10.3 ± 0.5 pA, the mean noise of the recordings at −65 mV, were characterized as a failure. In BA PNs, every light stimulus produced an AMPAR-mediated oEPSC and, hence, zero failures or a failure rate, i.e. number of failures divided by total number of light stimuli, of 0%. The failure rate was significantly higher in CeL neurons with 14.58 ± 3.42% (Mann-Whitney rank sum test: p = 4.7E-4) (Fig. 2H). NMDAR-mediated oEPSCs were significantly smaller in CeL as compared to BA neurons. The mean amplitude of the NMDAR component was 35.80 ± 4.68 pA in CeL and 153.52 ± 34.89 pA in BA neurons (unpaired t-test: p = 8.2E-3) (Fig. 2G). The failure rate of the NMDAR component of the oEPSC was 5.25 ± 2.55% in CeL neurons and 0% in BA PNs (Fig. 2H). The AMPAR/NMDAR ratio, an indicator of basal synaptic strength, was calculated as the quotient of the mean oEPSC amplitude at −65 mV divided by the mean oEPSC amplitude at +40 mV and did not differ significantly between CeL and BA neurons with values of 1.64 ± 0.24 and 1.39 ± 0.29, respectively (Fig. 2I). Interestingly, some (n = 5/21) CeL neurons that did not receive AMPAR-mediated dMT input at −65 mV showed an NMDAR-mediated component of the oEPSC when clamped at +40 mV (Fig. 2E,F). These so called silent synapses were not observed in BA (Fig. 2D,E)^25^. The paired-pulse ratio, calculated on the basis of two AMPAR-mediated oEPSC amplitudes in response to two light stimuli at an interval of 100 ms (PPR = A2/A1), was significantly smaller for dMT-BA as compared to dMT-CeL synapses indicating a higher initial probability of neurotransmitter release at dMT-BA synapses (Fig. 2J). Together, the data indicate that CeL neurons receive smaller and less reliable dMT inputs as compared to BA PNs. Furthermore, dMT and CeL display weaker apparent connectivity as compared to dMT and BA. Since BA PNs are intermingled with interneurons (BA INs) in the BA^18^, we investigated whether dMT inputs evoke feedforward inhibition onto BA PNs. At a holding potential of −45 mV we recorded monosynaptic oEPSCs with a mean amplitude of −119.62 ± 30.68 pA but never polysynaptic oIPSCs in the same cells (n = 11 cells/2 mice) (Supplementary Fig. S2D–E).

### µ-opioids attenuate dMT synaptic inputs to BA PNs and CeL neurons

**Figure 3:**
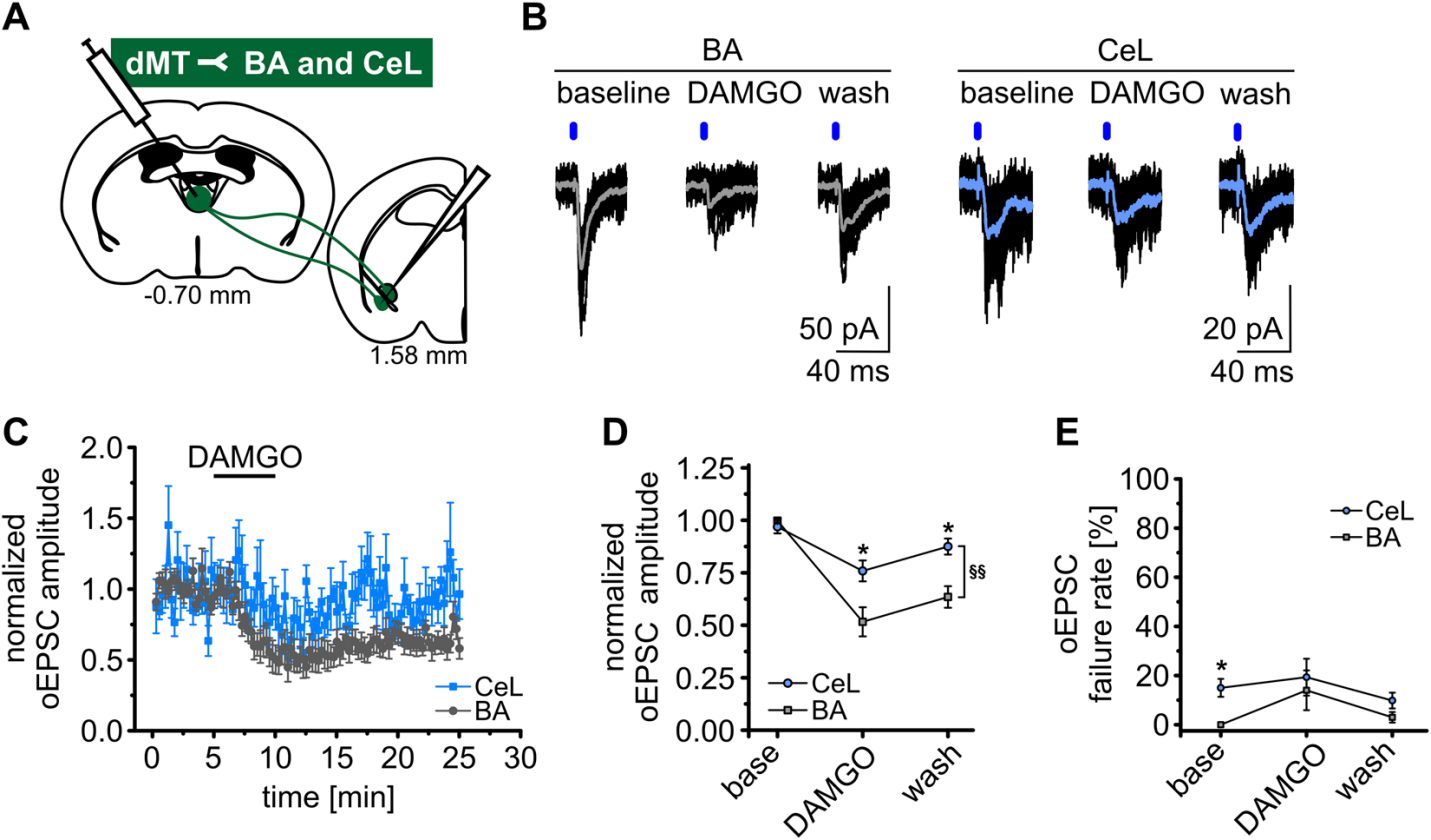
MOR stimulation attenuates dMT synaptic inputs to BA PNs and CeL neurons. (**A**) dMT neurons were transduced with AAV2-ChR2-eYFP. Optogenetic stimulation and patch-clamp recordings of BA PNs and CeL neurons were performed six weeks later. (**B**) Representative traces of oEPSCs recorded in BA and CeL neurons at a holding potential of −65 mV. Depicted are oEPSC amplitudes during baseline, DAMGO bath application and wash. Blue bars indicate the light stimulus. (**C**) Mean normalized oEPSC amplitude in BA PNs (n = 11 cells/7 mice) and CeL neurons (n = 7 cells/7 mice). DAMGO application is indicated by the horizontal line and lead to a decrease in the normalized oEPSC amplitude in BA PNs and CeL neurons. (**D,E**) Quantification of the mean normalized oEPSC amplitude (**D**) and failure rate (**E**) in BA and CeL neurons before (baseline), during maximal DAMGO response and following washout. The attenuating effect of DAMGO on the oEPSC amplitude was significantly stronger at dMT-BA synapses as compared to dMT-CeL synapses (**D**).

We next explored whether the projections from dMT to BA or CeL are differentially modulated by µ-opioids at the synaptic level (Fig. 3A). AMPAR-mediated oEPSCs were recorded at −65 mV, as described above and exhibited a mean amplitude of −127.72 ± 27.60 pA in BA neurons, and of −39.32 ± 6.13 pA in CeL neurons under baseline conditions. Bath application of 250 nM DAMGO for 5 min led to a reduction in the mean oEPSC amplitude (Fig. 3B,C). oEPSC amplitudes were normalized to baseline to quantify the effect size of MOR stimulation in BA versus CeL neurons. The mean oEPSC amplitude in BA PNs was significantly reduced to 0.52 ± 0.07 by DAMGO and 0.63 ± 0.05 after wash. In CeL neurons the normalized oEPSC amplitude was 0.76 ± 0.05 after DAMGO and 0.87 ± 0.04 after wash (repeated measures ANOVA: F_(2,32)_ = 8.45, p = 1.13E-3; *post hoc* test: baseline_BA_ vs DAMGO_BA_, p = 2.23E-14; wash_BA_ vs baseline_BA_, p = 1.45E-7; baseline_CeL_ vs. DAMGO_CeL_, p = 1.87E-2; DAMGO_CeL_ vs DAMGO_BA_, p = 2.41E-2; wash_CeL_ vs wash_BA_, p = 2.63E-2) (Fig. 3D). The oEPSC failure rates at dMT-CeL and dMT-BA synapses were not significantly influenced by DAMGO (Fig. 3E). As evident from Fig. 3C, the DAMGO-induced inhibition did not fully reverse upon washout. We can, however, exclude a rundown of oEPSC amplitudes with time, since BA PNs recorded in the absence of DAMGO exhibited constant oEPSC amplitudes during the same time course of recording (Fig. S3A). oEPSC amplitudes were quantified at time points that correspond to those used to asses DAMGO effects in Fig. 3C and revealed no significant differences across time (one way ANOVA: F_(2,39)_ = 0.781; p = 0.465) (Fig. S3B). Moreover, during DAMGO-induced inhibition of dMT inputs to BA PNs, we added the opioid receptor antagonist Naloxone (1 µM) to the bathing solution (Fig. S3C). In the +Naloxone group, oEPSC amplitudes were significantly reduced to 0.51 ± 0.04 after DAMGO and reversed to 0.90 ± 0.10 during washout in 1 µM Naloxone (repeated measures ANOVA: F_(2,36)_ = 4,06, p = 2.57E-2; *post hoc* test: baseline_+Nalox_ vs DAMGO_+Nalox_, p < 0.0001; DAMGO_+Nalox_ vs wash_+Nalox_, p < 0.0001; wash_+Nalox_ vs baseline_+Nalox_, p = 0.45; wash_+Nalox_ vs wash_-Nalox_, p = 8.9E-3) (Fig. S3D). Together, these results provide evidence for a DAMGO-induced inhibition of dMT inputs to both BA and CeL mediated via MORs, with weaker modulatory influence on dMT-CeL synapses as compared to dMT-BA synapses.

### Activation of MORs suppresses dMT-driven feedforward excitation of CeM neurons

**Figure 4:**
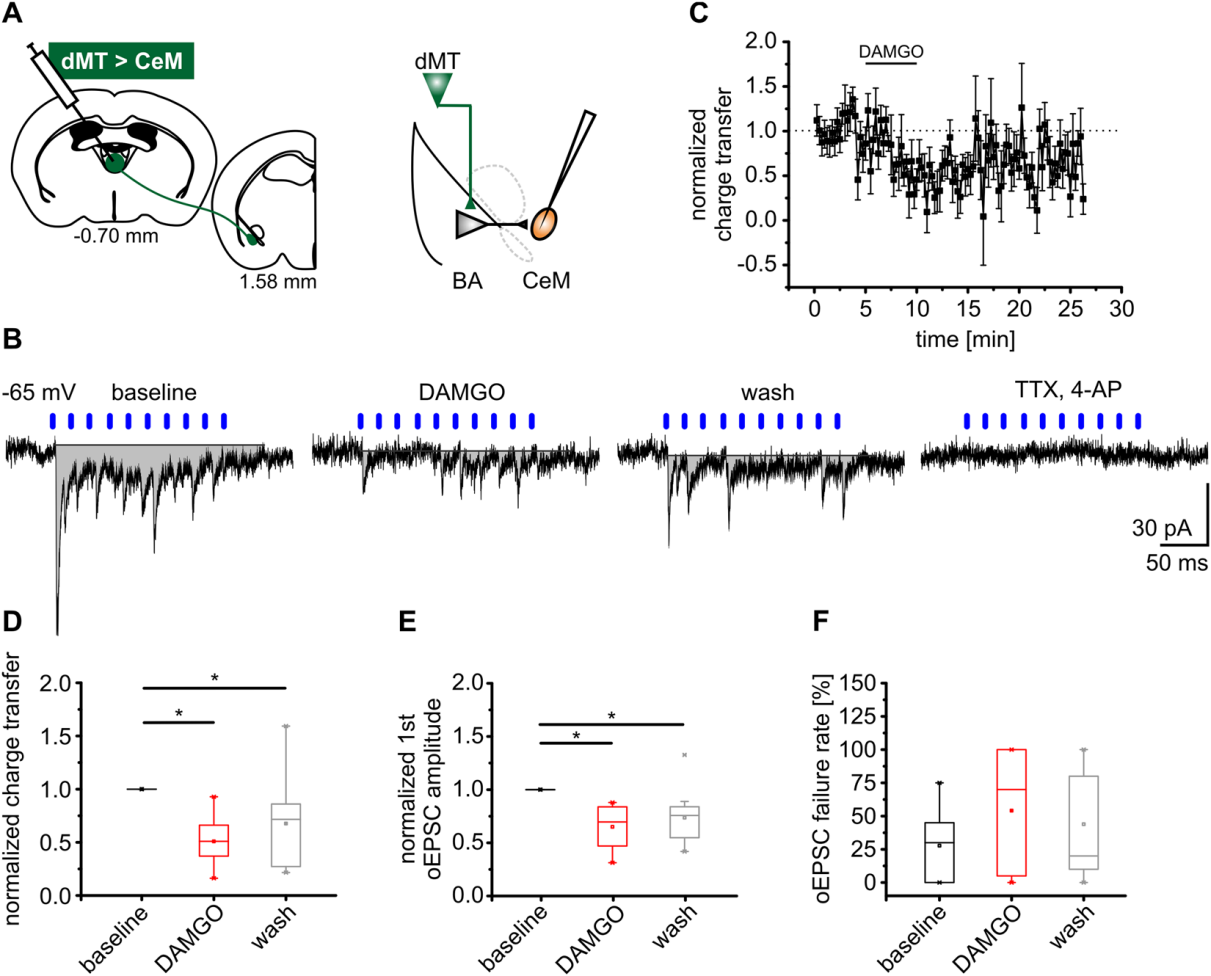
The MOR agonist DAMGO attenuates dMT-driven feedforward excitation of CeM neurons. (**A**) dMT neurons were transduced using AAV2-ChR2-eYFP and optogenetic experiments were performed six weeks later. dMT fibers were light-stimulated in BA and oEPSCs recorded in CeM neurons. (**B**) Representative traces recorded in CeM neurons at a holding potential of −65 mV in presence of GABA_A_ and GABA_B_ receptor antagonists. 50 Hz light stimulation of dMT afferents in BA (10 pulses indicated by blue bars) evoked multiple oEPSCs in CeM neurons. The total current across the membrane during the period of light-stimulation was estimated by calculating the area under the curve (= charge transfer; grey). Application of DAMGO reduced the charge transfer with a partial recovery during wash. 1 µM TTX and 100 µM 4-AP abolished oEPSCs. (**C**) Time course of the normalized charge transfer. The period of substance application is indicated by the horizontal line. (**D–F**) Quantification of the normalized charge transfer (**D**), the normalized amplitude of the first oEPSC (**E**), and the failure rate of the first oEPSC (F) (n = 11 cells/7 mice).

The dMT provides extensive excitatory input to BA PNs which is attenuated by MOR activation. Further on, BA PNs send excitatory projections to the CeM, mediating fear-related signals via projections to hypothalamic and brainstem nuclei^18,19^. We hypothesized that dMT afferents in BA evoke feedforward excitation in CeM neurons, which is sensitive to µ-opioidergic modulation. Neurons were recorded in CeM and a train of 10 light stimuli at 50 Hz was applied to dMT afferents in the BA in order to evoke polysynaptic transmission onto CeM neurons (Fig. 4A). Among all recorded CeM neurons, 51% received dMT-driven feedforward excitatory input. In fact, multiple oEPSCs occurred in CeM neurons recorded at a holding potential of −65 mV. These multiple oEPSCs were attenuated in the presence of DAMGO (Fig. 4B). In 5/11 recordings, TTX and 4-AP were applied after wash out of DAMGO and blocked oEPSCs in all cases (Fig. 4B), indicating a polysynaptic transmission. The total synaptic current across the membrane during the whole period of light-stimulation was expressed as a charge transfer of −1353 ± 287 fC during baseline. Normalized to baseline, the charge transfer was significantly reduced to 0.51 ± 0.06 by DAMGO and recovered to 0.68 ± 0.12 after wash out (one way ANOVA: F_(2,30)_ = 10.44, p = 3.62E-4; *post hoc* test: baseline vs DAMGO, p = 2.90E-4; wash vs baseline, p = 1.79E-2) (Fig. 4B–D). In addition to the charge transfer, the amplitude and failure rate of the first oEPSC were analyzed. The amplitude was significantly attenuated to 0.65 ± 0.08 by DAMGO and returned to 0.74 ± 0.08 of the baseline value after wash (one way ANOVA: F_(2,26)_ = 8.80, p = 1.21E-3; *post hoc* test: baseline vs. DAMGO, p = 1.82E-3; wash vs baseline, p = 1.30E-2) (Fig. 4B,E). The mean failure rate of 27.7 ± 8.0% during baseline was not significantly changed by DAMGO (Fig. 4F). These results indicate that an attenuation of dMT synaptic inputs to BA by the MOR agonist DAMGO subsequently reduces feedforward excitation of CeM neurons.

## Discussion

The dMT, specifically the PVT, is a key node to balance competing behavioral demands to reward and danger^26^. Considering inputs and outputs of the PVT, a general view by Kirouac (2015) states that, while approach behavior is mediated by PVT projections to the NAc, PVT projections to the amygdala may regulate aversive or defensive behavioral states. The amygdala, in a similar way, processes both positive and negative emotional stimuli where negative valence is predominantly mediated by basolateral amygdala projections to the CeM^27^. Here, we provide evidence of a dMT-amygdala excitatory synaptic circuit that may be involved in signaling aversive behavioral states and demonstrate a significant neuromodulatory role for the µ-opioid system in the respective network.

Projections of the dMT to the CeL have been studied in great detail and were implicated in mediating fear expression^22,28,29^. The role for dMT-BA projections, however, remains less clear. Our data suggest that the BA is the dominant target area for excitatory dMT projections in the amygdala, and reveal weaker apparent connectivity of dMT and CeL. Importantly, our results indicate that dMT-driven BA activity translates into CeM synaptic activation to a considerable degree. Via this circuit, the dMT could activate CeM neurons which in turn mediate aversive behavioral responses through projections to brainstem and hypothalamic nuclei^18^. dMT inputs to the CeL may act in a similar way. Afferents from the dMT, i.e. the PVT, preferentially target corticotropin-releasing hormone and somatostatin (SOM)-expressing neurons and only to a lesser extent protein kinase C δ (PKCδ)-expressing neurons in the CeL^22,30^. Based on the disinhibitory circuit model proposed by Haubensak *et al*.^31^ and Ciocchi *et al*.^32^ where SOM expressing CeL neurons inhibit PKCδ expressing CeL neurons, thereby disinhibiting CeM activity, dMT inputs to the CeL would predominantly increase aversive behavioral responses.

Our injections targeted the PVT and sometimes extended to the neighboring paratenial nucleus (PT), medial part of the mediodorsal nucleus (MDm), and central nucleus (CM). For the PT, the connectivity largely parallels that of the PVT and also anatomical and functional characteristics of MDm and CM have been shown to be similar to that of the PVT^9^. Labeled dMT axonal projections appeared densest in the aBA and one may speculate that the target neurons of dMT projections in the BA overlap with a population of magnocellular neurons in the aBA that have been shown to be recruited by negative stimuli (e.g. shocks) to induce aversive behaviors^33^. Neurons in the posterior part of the BA are, in contrast, engaged in appetitive behaviors following positive stimuli (e.g. sucrose)^33^. Interestingly, we found that light-stimulation of dMT afferents in the amygdala well evoked monosynaptic oEPSCs but did not elicit polysynaptic oIPSCs in BA PNs. This corresponds nicely to ultrastructural data proving that PVT axons mostly contact BA PNs but almost no BA INs. Amir *et al*. (2019) hypothesized that little feedforward inhibition counteracts the PVT’s excitatory influence on BA PNs^34^, which is in line with our results demonstrating that light stimulation of dMT afferents in the BA markedly drives feedforward excitation of CeM neurons. We cannot exclude that excitatory neurons of the endopiriform cortex or basomedial nucleus of the amygdala receive dMT input and contribute to CeM feedforward excitation. However, we show that BA PNs receive substantial input from dMT and, thus, likely mediate feedforward excitation of CeM neurons in large part. Furthermore, we find here that dMT and BA display higher apparent connectivity as compared to dMT and CeL and that CeL neurons receive smaller and less reliable dMT inputs as compared to BA PNs. The increased strength of synaptic transmission at dMT-BA synapses may likely be explained by a stronger innervation of the BA by dMT afferents as compared to CeL. Further, the paired-pulse ratio was significantly lower at dMT-BA synapses as compared to dMT-CeL synapses indicating an increased initial probability of neurotransmitter release. Moreover, an increased number of AMPARs in the postsynaptic membrane could account for the observed differences. The AMPAR/NMDAR ratio, however, did not differ significantly between dMT-CeL and dMT-BA synapses and thus, did not allow for a clear interpretation of postsynaptic AMPAR occupancy. Since we did not distinguish between SOM or PKC-δ expressing CeL neurons in our analysis, it is possible that responses of neurons receiving strong or weak dMT input^22,30^ were averaged leading to an apparent overall reduced synaptic efficacy in CeL compared to BA. Notably, we observed silent synapses in the CeL which may be activated by increased synaptic input and subsequently, act as mediators of plasticity^25^.

Our data demonstrate that MOR stimulation substantially downregulates excitatory activity in dMT-amygdala circuits, emphasizing an important neuromodulatory role for the µ-opioid system. While there is good evidence for dense MOR expression in dMT neurons^5–7^, exact cellular and synaptic mechanisms of µ-opioidergic modulation in the dMT-amygdala circuitry remained unresolved. Since Penzo *et al*.^22^ stated that dMT neurons projecting to CeL and BA constitute non-overlapping populations, we studied whether MOR influence on these populations differs. We show here that both CeL and BA projecting dMT neurons are hyperpolarized by activation of MORs. The hyperpolarization was accompanied by a reduction in apparent input resistance suggesting mediation by an increase in potassium conductance. In fact, MOR signaling is known to activate GIRKs in several brain areas, including PVT^20,21,35^. It is of note, that our analyses have focused on neurons in a middle part of the dMT/PVT (Bregma −0.7 to −0.9 according to Paxinos and Franklin “The mouse brain in stereotaxic coordinates” second edition, 2001) which is highly interconnected with the amygdala. Neurons in more anterior portions of the PVT/dMT, in contrast, send only sparse fibers to the BA, and previous studies have suggested different roles for anterior (aPVT) and posterior PVT (pPVT) in motivated behaviors^8,12^. Although MOR gene expression (*Oprm1*) seems to be uniform along the anteroposterior axis of the dMT/PVT^5–7^, an interesting question for future studies is whether aPVT and pPVT are differentially modulated by opioids.

In addition to cellular excitability, MORs have been shown to modulate synaptic transmission in a variety of brain regions such as the locus coeruleus, PAG, and amygdala^19,35^. The present findings show that MOR signaling downregulates dMT excitatory synaptic inputs to BA PNs and CeL neurons, with a stronger impact at dMT-BA synapses. While here DAMGO was used as MOR agonist to assess the impact on synaptic transmission between dMT and amygdala, different agonists should be used to address ligand-based signaling effects in these pathways in future approaches. Previous studies have shown that MORs localized in synaptic terminals mediate the inhibition of voltage-activated calcium channels^20^. Thus, the attenuation of oEPSCs in BA PNs and CeL neurons may be linked to MOR-mediated inhibition of presynaptic calcium channels which leads to a reduction in glutamate release. Importantly, we prove that MOR stimulation attenuates dMT-driven feedforward excitation of the CeM. Since we demonstrated in a previous study that BA projections to CeM neurons are not modulated by MORs^19^, the attenuation of CeM feedforward excitation is most likely attributed to the reduction of synaptic transmission at dMT-BA synapses. Our findings are part of a rather complex dMT-amygdala network with many-faceted actions of MORs. Besides the dMT-BA-CeM pathway, the activity of CeM output neurons is also regulated through a BA-driven feedforward inhibitory circuit via intercalated neurons of the amygdala which we have described before^19^. We contrast MOR modulatory effects in these circuits and provide a schematic presentation of the network in Fig. S4. In conclusion, MORs are important negative modulators of dMT-amygdala excitatory synaptic circuits and could thereby, ameliorate aversive or defensive behavioral states emerging during stress, physical pain or social rejection. In fact, enkephalinergic neurotransmission in the basolateral amygdala has been shown to attenuate stress-induced neuroendocrine, autonomic, and anxiety-like responses^36^. Eventually, the aversive state and anxiety-like symptoms during withdrawal in opioid-dependent subjects, where drug-tolerance occurs and opioid receptors are downregulated^37^, may be related to a lack of µ-opioidergic inhibition in dMT-amygdala circuits. This might implicate an important role for MOR signaling in dMT-amygdala circuits in comorbid addiction and anxiety disorders.

## Methods

Adult, 9–16 weeks old C57BL/6 J mice were used and all animal experiments were performed in accordance with European regulations on animal experimentation (Directive 2010/63/EU of the European parliament and the council). All protocols were approved by the local authorities (Bezirksregierung Münster and the ‘Landesamt für Natur, Umwelt und Verbraucherschutz Nordrhein-Westfalen’). A cholera toxin subunit B Alexa Fluor 488 conjugate (CTB-Al488; Life Technologies) was used for retrograde tracing experiments. Viral transduction of neurons in the dMT was performed using recombinant adeno-associated viruses of serotype 2 (rAAV2) carrying a fusion construct of the coding sequences of the light-activated Channelrhodopsin 2 and enhanced yellow fluorescent protein (eYFP) under the control of the human synapsin promoter (rAAV2-hSyn-hChR2-eYFP; University of North Carolina Vector Core). rAAV2s were produced using pAAV-hSyn-hChR2(H134R)-EYFP which was a gift from Karl Deisseroth (Addgene plasmid # 26973). Acute brain slices were maintained at 31 °C during whole-cell recordings performed according to standard procedures. Electrophysiological data were acquired at a sampling rate of 10 kHz with an EPC-10 patch-clamp amplifier in combination with the software Pulse (HEKA), and analyzed offline in Clampfit 9.2 (Molecular Devices). In the electrophysiological experiments, the specific MOR agonist D-Ala^2^-NMe-Phe^4^-Gly-ol^5^-enkephalin (DAMGO, R&D Systems) was bath-applied for 5 min at a flow rate of 3-3.5 mL/min and a concentration of 250 nM. The non-selective opioid receptor antagonist Naloxone (Naloxone hydrochloride, R&D Systems) was bath-applied at a concentration of 1 µM. ChR2-eYFP expressing fibers in the amygdala were stimulated using microscope-objective coupled LEDs (460 nm, 45 mW, Prizmatix) and oEPSCs were recorded in voltage-clamp mode. To study feedforward excitation of CeM neurons, dMT axonal projections in the BA were stimulated with 10 light-pulses at 50 Hz. Data are shown as box plots. The box represents the first (25%) and third (75%) quartile, the whiskers represent the 5% and 95% percentiles. The band inside the box represents the median, the square represents the mean. In scatter plots, whiskers represent the standard error. The number of experiments is given as number of recorded neurons/number of animals. The detailed experimental procedures are provided in the Supplementary Information.

## Supplementary information

Supplementary Information

## Data Availability

The datasets generated and/or analyzed during the current study are available from the corresponding author on reasonable request.
