# Supplementary material for: µ-opioid receptor-mediated downregulation of midline thalamic pathways to basal and central amygdala
Source: Sci Rep. 2019 Nov 28;9:17837. doi: 10.1038/s41598-019-54128-8 (PMC6882837; doi:10.1038/s41598-019-54128-8)
Supplement: Supplementary file 1 — Supplementary Information [file 41598_2019_54128_MOESM1_ESM.pdf]

Supplementary Information

μ-opioid receptor-mediated downregulation of midline thalamic pathways to basal and central amygdala

Goedecke L.*, Bengoetxea X., Blaesse P., Pape H.-C., and Juengling K.

Affiliation: Institute of Physiology I, Westfaelische Wilhelms-Universitaet Muenster, Germany

*Correspondence: lena.goedecke@ukmuenster.de

Supplementary Figures

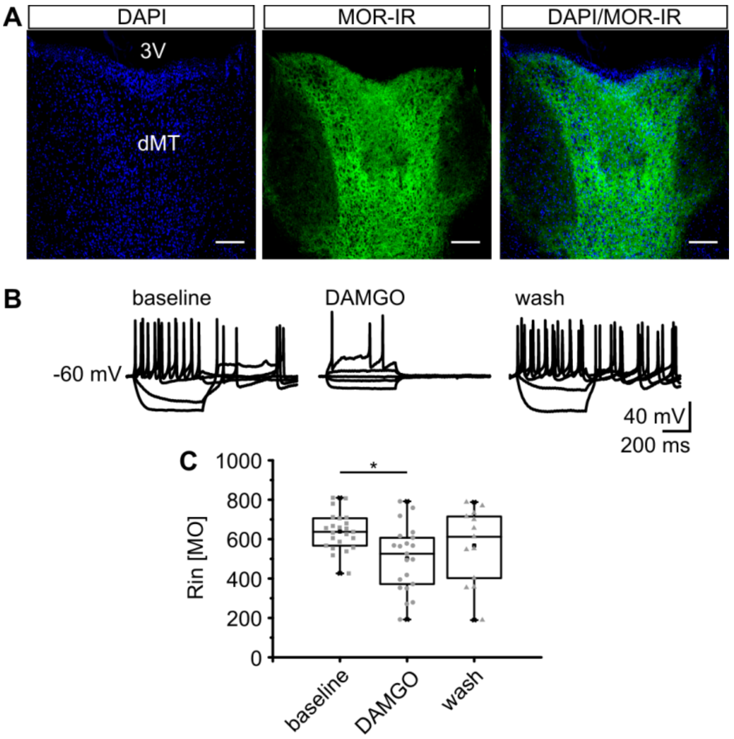

Supplementary Figure 1. MORs in the dMT.

(A) DAPI staining illustrates distribution of cell nuclei in the dMT. MOR labeling reveals a strong and uniform MOR-IR in the dMT. Scale bars represent 100 µm.

(B) Representative traces of responses to hyper- and depolarizing current steps of increasing amplitude (-60 pA, -20 pA, 0 pA, +20 pA, +60 pA) from -60 mV in a dMT neuron during baseline, maximal DAMGO effect and wash.

(C) The apparent input resistance was calculated from the voltage-deflection in response to the -60 pA hyperpolarizing current step. During maximal effect the cell was manually clamped back to -60 mV to exclude a possible influence of voltage-dependent conductances on the input resistance (n = 23 cells/9 mice). MOR activation reduced the apparent input resistance of dMT neurons projecting to BA and CeL.

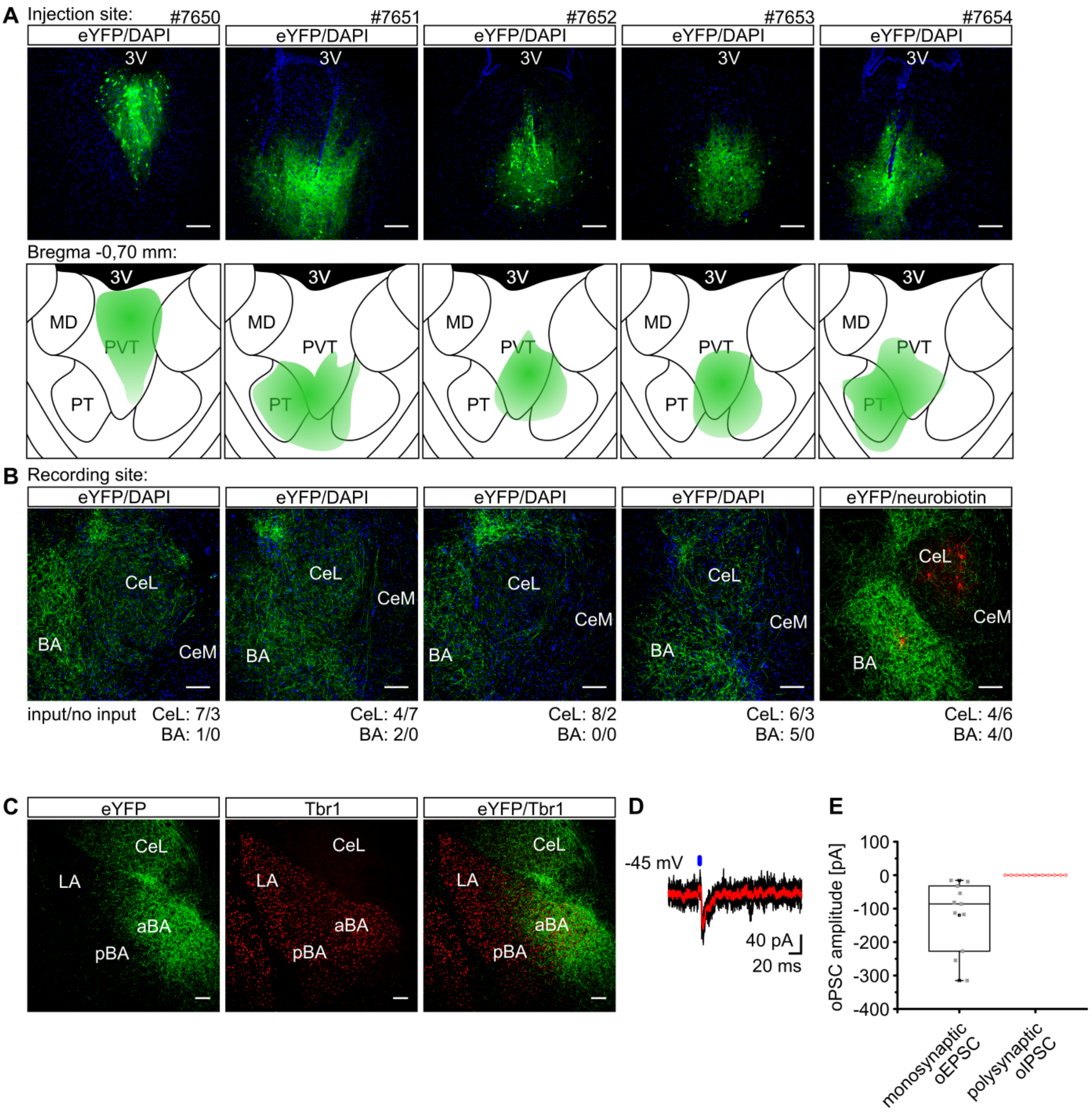

Supplementary Figure 2. Characterization of dMT-BA and dMT-CeL projection patterns and synaptic transmission.

(A) Histology and schematic presentation of AAV2-ChR2-eYFP injection sites in the dMT in five mice included in the analysis (#7650-7654).

(B) ChR2-eYFP expressing dMT projections in BA and CeL. For each mouse, recordings were initiated in that brain slice exhibiting most prominent expression of labeled fibers which is displayed here. Number of recorded neurons that received AMPAR-mediated oEPCS versus non-responding cells in CeL and BA.

(C) Tbr1-IR marks PNs of cortical origin and thereby distinguishes basolateral (LA and BA) from central amygdala (CeL and CeM). ChR2-eYFP expressing dMT projections are

especially dense in the CeL and anterior portion of the BA (aBA) but not in the LA or posterior BA (pBA).

(D) Representative trace of dMT-driven PSCs in BA PNs at a holding potential of -45 mV.

(E) Absolute oPSC amplitudes: Light stimulation of dMT projections in the BA evoked monosynaptic oEPSC in all cells but never polysynaptic oIPSCs (n = 11 cells/2 mice).

Scale bars represent 100 µm.

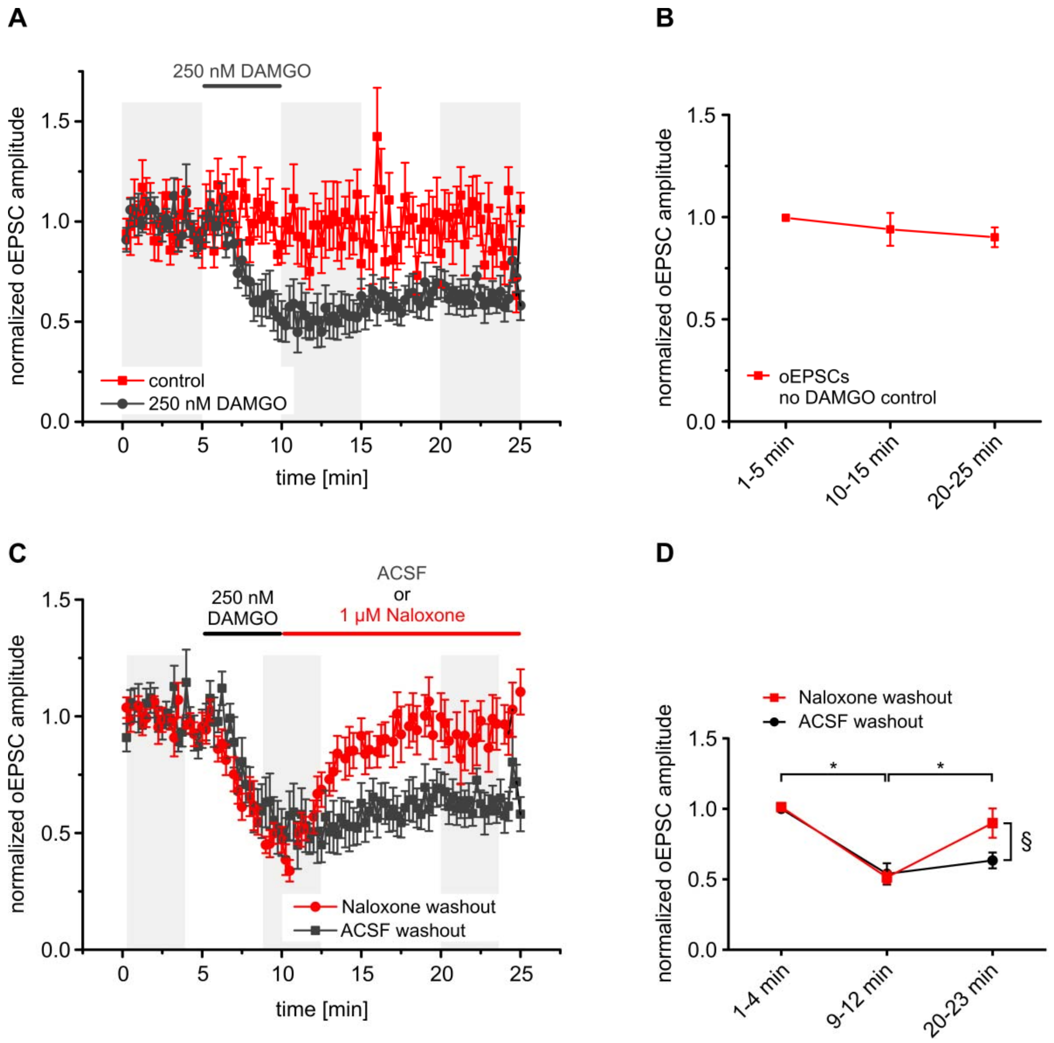

Supplementary Figure 3. The DAMGO-induced inhibition of dMT inputs to BA PNs is dependent on opioid receptors.

(A) In the absence of DAMGO, oEPSC amplitudes are constant over the time course of the recording. Mean normalized oEPSC amplitudes in BA PNs under control conditions without DAMGO application (n = 14 cells/5 mice) in comparison to oEPSCs in BA PNs (n = 11 cells/7 mice) before, during, and after DAMGO application (horizontal line). The data set of oEPSCs in BA PNs under DAMGO (grey) is the same as shown in Fig. 3C.

(B) Quantification of the mean normalized oEPSC amplitudes in BA PNs under control conditions at 1-5 mins, 10-15 mins, and 20-25 mins. Control BA PNs did not exhibit a reduction in oEPSC amplitude as it was seen during maximal DAMGO effect (10-15 mins) and washout (20-25 mins) for BA PNs after DAMGO application.

(C) DAMGO-induced reduction in oEPSC amplitudes is fully reversed by application of the non-selective opioid receptor antagonist Naloxone. Mean normalized oEPSC amplitudes in

BA PNs during baseline, DAMGO application, and washout in 1 µM Naloxone in comparison to washout in ACSF (data set in grey from Fig. 3C and Fig. S3A).

(D) Quantification of the mean normalized oEPSC amplitudes in BA PNs at 1-4 mins, 9-12 mins, and 20-23 mins. oEPSC amplitudes in BA PNs were attenuated following DAMGO and fully recovered during washout in Naloxone (n = 10 cells/3 mice).

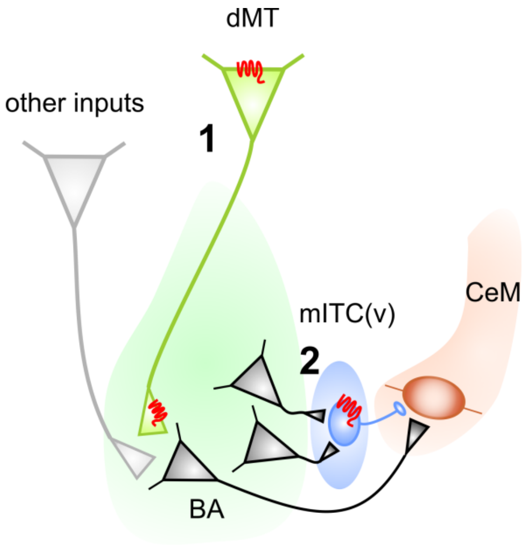

Supplementary Figure 4. Schematic presentation of two MOR modulated pathways projecting onto CeM output neurons.

In a very simplified scenario, two independent pathways project onto a CeM output neuron. The first one (1) consists of glutamatergic dMT inputs that drive feedforward excitation via BA principal neurons. In the second pathway (2), glutamatergic BA principal neurons, which might differ from BA PNs of the first circuit, project onto intercalated cells of the medioventral cluster (mITC(v)), triggering feedforward inhibition of the CeM neuron [19]. A broad activation of MORs in both pathways should reduce both, feedforward excitation and inhibition, with limited net effect in the CeM neuron. In the CeM output neuron, general synaptic activity would be reduced without a shift of the excitation/inhibition ratio. This would only be valid for this reductionist scheme with only one driving input in both pathways and the assumption that inhibitory and excitatory inputs have comparable magnitude. If one considers that multiple glutamatergic inputs can drive feedforward inhibition via mITC(v)s and feedforward excitation via BA PNs, the situation might be very different. In this scenario, the somatic, inhibitory effect of MOR activation in mITC(v)s (2) might be much more effective, switching off feedforward inhibition driven by multiple sources. On the other hand, feedforward excitation might be less affected, since not all inputs on BA PNs are modulated by MOR (1). In that pathway, MOR activation reduces the excitatory drive of a fraction of inputs, leaving others unaffected. In that scenario, broad, synchronized MOR activation might shift the excitation/inhibition ratio in CeM neurons in favor of excitation. Whether such a synchronized MOR activation occurs *in vivo* is not clear, but it seems more likely that MOR activation in the different pathways is much more diverse, depending e.g. on the behavioral context and activated sources of endogenous opioids.

Supplementary Methods

Animals and Ethical Approval.

Adult C57BL/6J mice used had access to food and water ad libitum at a 12 h light/dark cycle with lights on at 6:00 am. All animal experiments were performed in accordance with European regulations on animal experimentation (Directive 2010/63/EU of the European parliament and the council) and protocols were approved by the local authorities (Bezirksregierung Münster and the 'Landesamt für Natur, Umwelt und Verbraucherschutz Nordrhein-Westfalen').

Retrograde Tracing and Viral Transduction.

For retrograde tracing experiments, CTB-Al488 was diluted to a working concentration of 0.5 % (w/v) in 0.1 M phophate-buffered saline (PBS). 100 nL CTB-Al488 was delivered locally into the CeL or BA of C57BL/6J mice by stereotaxic injection. Viral transduction of neurons in the dMT was performed using 300 nL rAAV2-hSyn-hChR2-eYFP. Coordinates for CeL injection were -1.65 mm anteroposterior (AP), ±3.00 mm mediolateral (ML), and 3.90 mm dorsoventral (DV) according to bregma. BA coordinates were -1.65 mm AP, ±3.30 mm ML, and 4.10 mm DV. dMT coordinates were -0.60 mm AP, 0.30 mm ML, and 3.15 mm DV. dMT injections were conducted in a 5° angle ML to prevent damage of the *sinus sagittalis superior*. Prior to stereotaxic surgery, mice were anesthetized with an intraperitoneal injection of sodium pentobarbital (50 mg/kg), along with subcutaneously injected Carprofen (5 mg/kg, Rimadyl, Pfizer) as an analgesic. The surgery was performed according to standard protocols. A 10 μl Hamilton syringe with a 33 gauge beveled needle was moved with a micromanipulator, and served to deliver the retrograde tracer or rAAV into the brain. Following manual injection, the needle was left in place for 10 min and was then carefully and slowly pulled out. 3-4 days following the surgical procedure, electrophysiological recordings were performed on retrogradely labeled neurons in the dMT. Optogenetic experiments were carried out 5-7 weeks following surgery.

Electrophysiology and Optogenetic Stimulation.

Mice were anesthetized in isoflurane (2.5 % in O_2, AbbVie) and decapitated. After surgical extraction of the brain, 300 μm thick coronal slices were cut on a vibratome (VT 1200 S, Leica). For whole-cell patch-clamp recordings, brain slices were placed in a submersion chamber and perfused with artificial cerebrospinal fluid (aCSF) composed of (in mM)

120 NaCl, 2.5 KCl, 1.25 NaH_2PO_4, 2 $MgSO_4$, 2 $CaCl_2$, 22 $NaHCO_3$, and 25 glucose at 32°C. The pH was adjusted to 7.35 by gassing with carbogen. The ion channel blockers TTX (1 μM, Biotrend), 4-AP (100 μM), DNQX (10 μM), AP-5 (50 μM), gabazine (25 μM), and CGP55845 (10 μM) were added to the aCSF when required (Abcam). Borosilicate glass capillaries (Harvard Apparatus) were used as recording pipettes (2-4 MΩ) and filled with an internal solution containing (in mM) 10 NaCl, 88 potassium gluconate, 20 potassium citrate, 10 HEPES, 3 BAPTA, 15 phosphocreatine, 1 $MgCl_2$, 0.5 $CaCl_2$, 3 Mg-ATP, and 0.5 Na-GTP. All electrophysiological data were acquired at a sampling rate of 10 kHz with an EPC-10 patch-clamp amplifier in combination with the software Pulse (HEKA) and analyzed offline in Clampfit 9.2 (Molecular Devices). The series resistance was monitored throughout the recordings and data were discarded when sudden changes occurred. The specific MOR agonist D-Ala^2-NMe-Phe^4-Gly-ol^5-enkephalin (DAMGO, R&D Systems) was bath-applied for 5 min at a flow rate of 3-3.5 mL/min and a concentration of 250 nM.

Membrane potential and input resistance of dMT neurons. Retrogradely traced neurons in the dMT after BA or CeA injection of CTB-Al488 were identified by their somatic fluorescence. The membrane potential was recorded in current-clamp mode in aCSF containing DNQX and AP-5 to block glutamatergic, and gabazine, and CGP55845 to block GABAergic synaptic transmission. To ensure comparable ionic driving forces for all dMT neurons, the membrane potential was adjusted to approximately -60 mV before recording baseline. Current-voltage relationships (current pulse duration: 500 ms, maximal hyperpolarizing pulse: -60 pA, increment: 10 pA) were measured during baseline, maximal effect, and washout of DAMGO to assess changes in input resistance. During maximal effect, a direct current offset was applied to set back the membrane potential to -60 mV, in order to exclude voltage-dependent conductance contributing to changes in input resistance and thus, to isolate direct DAMGO-induced effects.

Optogenetically evoked excitatory postsynaptic currents in BA PNs and CeL neurons. In order to study dMT synaptic inputs to BA and CeL neurons, we used an optogenetic approach. ChR2-eYFP expressing axonal projections in the amygdala were stimulated using microscope objective-coupled LEDs (460 nm, 45 mW, Prizmatix). 10-20 light stimuli were applied at 15 s intervals and evoked excitatory postsynaptic currents (oEPSCs) in BA and CeL neurons recorded in voltage-clamp mode at a holding potential of -65 mV or produced failures. A failure was assigned, when the current deflection upon light stimulus was smaller than the noise of the recording. The mean noise for recordings at -65 mV in BA and CeL neurons was 10.3 ± 0.5 pA. Failures per total number of light stimuli (10-20) were expressed as failure rate

and remaining success oEPSC amplitudes were averaged. In some cells, the paired pulse ratio was assessed on the basis of two light stimuli at 10 Hz at an inter-stimulus interval of 15 s. Gabazine and CGP55845 were added to the aCSF, and TTX and 4-AP were present to block polysynaptic transmission [1]. After recording the AMPAR-mediated component of the oEPSCs at -65 mV, we clamped the same cell at a depolarized membrane potential of +40 mV to assess the NMDAR-mediated component of the oEPSC. To allow voltage clamp recordings at +40 mV, potassium currents were blocked using a cesium-based internal solution, containing (in mM) 5 4-AP, 120 $CsMeSO_4$, 1 EGTA, 10 HEPES, 20 tetraethylammonium chloride, 2 $MgCl_2$, 0.5 $CaCl_2$, 2 Na-ATP, and 0.5 Na-GTP. The mean noise of recordings at +40 mV was 19.5 ± 1.2 pA and smaller oEPSC amplitudes were considered failures. While the amplitude of the AMPAR-mediated oEPSC was always determined at the peak of the oEPSC at -65 mV, the amplitude of the longer-lasting NMDA component was determined at 95 ms post light-stimulation. At that point in time the AMPAR component had declined to zero. In addition to the absolute amplitudes of AMPAR- and NMDAR-mediated oEPSCs, their ratio was compared for CeL and BA neurons ($oEPSC_{-65mV}/oEPSC_{+40mV}$). Furthermore, dMT-CeL and dMT-BA apparent connectivity, i.e. the quotient of neurons receiving light-evoked input divided by the total number of neurons patched, was compared. In these experiments and when absolute oEPSC amplitudes and failure rates of dMT-BA and dMT-CeL synapses were compared, light-stimulation was kept constant at 1 ms pulse duration and 14 mW. The localization of recorded CeL and BA neurons and the corresponding size of the oEPSC amplitude were documented. Neurons were mapped at the tip of the recording electrode in the slice and pictures were overlayed manually. For *post hoc* identification, cells were filled with neurobiotin during some of the recordings. In order to study dMT-driven feedforward inhibition onto BA PNs, we further recorded BA PNs at a holding potential of -45 mV, positive to the reversal potential of GABA receptor-mediated inhibitory currents (E_{GABA}), to obtain biphasic responses composed of monosynaptic oEPSCs and disynaptic oIPSCs. A potassium gluconate internal solution and aCSF without synaptic blockers were used as described before. Following synaptic characterization, AMPAR-mediated oEPSCs were recorded in BA PNs and CeL neurons at -65 mV and the effect of MOR stimulation on mean normalized oEPSC amplitudes and failure rates was analyzed before DAMGO (baseline), during maximal DAMGO response and following wash. For each timepoint, responses to a series of 20 light stimuli were analyzed and success amplitudes were averaged. To confirm that DAMGO-induced effects are dependent on opioid receptors, the non-selective opioid receptor antagonist Naloxone (Naloxone hydrochloride, R&D Systems) was bath-applied

following DAMGO administration and during wash at a concentration of 1 µM in some experiments.

dMT-driven feedforward excitation of CeM. To study feedforward excitation of CeM neurons, dMT axonal projections in the BA were stimulated with 10 light-pulses at 50 Hz at an interval of 15 s. oEPSCs were recorded at -65 mV holding potential in an aCSF containing 1.2 mM $CaCl_2$ and 1 mM $MgSO_4$ to increase neuronal excitability. $GABA_{A/B}R$ blocker were contained in the aCSF and the recording pipette was filled with potassium-based internal solution. The total charge transfer during the multiple oEPSCs, as well as amplitude and failure rate of the first oEPSC were analyzed during baseline, maximal DAMGO effect, and washout in BA PNs.

Fluorescence detection and immunohistochemistry

For *post hoc* verification and documentation of the AAV2-ChR2-eYFP and CTB-Al488 injection and recording sites, acute slice preparations were fixed in 4 % paraformaldehyde in 0.15 M Na-phosphate buffer (pH 7.4) at 4°C for 24 hours and cryoprotected in 30 % sucrose in PBS. 40 µm thick sections were prepared and mounted in DAPI (4´,6-Diamino-2-phenylindole dihydrochloride)-containing Vectashield Mounting Medium (Vector Laboratories) without further immunohistochemical staining. When cells were filled with neurobiotin during the recording, 300 µm thick slices were kept and blocked for 1 hour in 3 % bovine serum albumin (BSA), 10 % goat serum, and 0.3 % Triton in PBS. After three wash steps in PBS, slices were incubated in blocking solution containing a streptavidin Alexa Fluor 546 conjugate (1:1,250, Life Technologies) for 2 hours.

Tbr1, a marker of glutamatergic neurons [2] was detected using a polyclonal rabbit anti-Tbr1 antibody (1 mg/ml; ab 31940; Abcam). Immunohistochemical stainings were performed on acute slices expressing ChR2-eYFP-positive fibers from the dMT in the BA and CeL. Following recordings, slices were fixed and cryoprotected as described above and 30 µm thick sections were cut. Sections were collected in 15 % sucrose/PBS, followed by three washes in PBS and were blocked for 1 hour. The anti-Tbr1 antibody was diluted 1:1,000 in blocking solution and incubation with agitation was carried out at 4°C overnight. After three wash steps in PBS, sections were incubated in carrier solution (0.3 % Triton, 1 % BSA, 1 % goat serum) containing the secondary antibody Alexa Fluor 546 goat anti-rabbit (2 mg/ml; 1:1,000 ; Life Technologies) for 2 hours at RT.

Immunohistochemical detection of the µ-opioid receptor was performed using a rabbit anti-MOR antibody (OPRM1 antibody; 24216, Origene Europe, Herford, Germany) [3]. In brief,

mice were anesthetized with isoflurane (5 %) and perfused transcardially with phosphate-buffered saline (pH 7.4; PBS), followed by 4 % paraformaldehyde (w/v) and 15 % saturated picric acid (v/v) in 0.15 M Na-phosphate buffer (pH 7.4) as fixative. After removal, brains were stored in fixative for 2 hours at RT. Brains were cryoprotected in a sucrose solution as described above and coronal sections (30 µm) were cut using a freezing microtome (Frigomobil 1205; Jung, Heidelberg, Germany). Sections were collected in 15 % sucrose/PBS, then blocked and incubated with the primary and secondary antibodies as described above. The anti-MOR antibody was diluted 1:1,000 in blocking solution. The secondary antibody goat anti-rabbit conjugated to AlexaFluor 488 was diluted 1:1,000 (LifeTechnologies) in carrier solution.

Cell nuclei were labelled using DAPI (1:1,000, Sigma-Aldrich) and after three additional washes in PBS, sections were mounted with Vectashield HardSet mounting medium (Vector laboratories, Burlingame, CA) or sections were directly mounted in DAPI-containing Vectashield Mounting Medium.

Imaging was performed on a laser scanning confocal microscope (Nikon eC1plus) equipped with a CFI75 LWD 16×/0.8NA or CFI Plan Fluor 10×/0.30NA objective (Nikon, Germany). Channels were imaged sequentially with 405 nm, 488 nm and/or 543 nm excitation in combination with adequate emission filters (450/30 nm, 515/30 nm, and 605/75, respectively) to prevent bleed-through. Images were further processed with ImageJ (http://rsb.info.nih.gov/ij).

Statistics.

Data are shown as box plots. The box represents the first (25 %) and third (75 %) quartile, the whiskers represent the 5 % and 95 % percentiles. The band inside the box represents the median, the square represents the mean. In scatter plots, whiskers represent the standard error. All data sets were tested for statistically significant outliers at a significance level of $\alpha = 0.05$ using the Grubb's test. Data were tested for Gaussian distribution using the Shapiro-Wilk test at a significance level of $\alpha = 0.05$. Within-group comparisons were done with paired student's t-test and differences between two different groups were analyzed with the unpaired t-test. Statistics for not normally distributed values were done using the Mann-Whitney rank sum test. Significant differences between three different groups were accessed using one-way ANOVA with Bonferroni *post hoc* test. To analyze significant differences between correlated samples and control for within-subject differences, repeated measures ANOVA with Bonferroni *post hoc* test was applied.

References

1. Cruikshank, S. J., Urabe, H., Nurmikko, A. V. & Connors, B. W. Pathway-specific feedforward circuits between thalamus and neocortex revealed by selective optical stimulation of axons. *Neuron* **65**, 230–245 (2010).

2. Waclaw, R. R., Ehrman, L. A., Pierani, A. & Campbell, K. Developmental Origin of the Neuronal Subtypes That Comprise the Amygdalar Fear Circuit in the Mouse. *J. Neurosci.* **30**, 6944–6953 (2010).

3. Arvidsson, U. *et al.* Distribution and targeting of a mu-opioid receptor (MOR1) in brain and spinal cord. *J. Neurosci.* **15**, 3328–3341 (1995).
